# Durability of Response to B‐Cell Maturation Antigen‐Directed mRNA Cell Therapy in Myasthenia Gravis

**DOI:** 10.1002/acn3.70167

**Published:** 2025-08-26

**Authors:** Nizar Chahin, Gregory Sahagian, Marc H. Feinberg, C. Andrew Stewart, Christopher M. Jewell, Metin Kurtoglu, Miloš D. Miljković, Tuan Vu, Tahseen Mozaffar, James F. Howard, the MG‐001 Study Group

**Affiliations:** ^1^ Department of Neurology Oregon Health and Sciences University Portland Oregon USA; ^2^ Neurology Center of Southern California San Diego California USA; ^3^ SFM Research Boca Raton Florida USA; ^4^ Cartesian Therapeutics Frederick Maryland USA; ^5^ Department of Neurology University of South Florida Tampa Florida USA; ^6^ Department of Neurology University of California Irvine California USA; ^7^ Department of Neurology University of North Carolina at Chapel Hill Chapel Hill North Carolina USA

**Keywords:** BCMA, CAR‐T, Descartes‐08, myasthenia gravis, autoimmune

## Abstract

**Objective:**

We report the 12‐month follow‐up outcomes from a Phase 2 clinical trial (NCT04146051) evaluating Descartes‐08, a BCMA‐directed RNA chimeric antigen receptor T‐cell (rCAR‐T) therapy for refractory generalized myasthenia gravis (MG). These findings provide insight into the potential applicability of BCMA‐targeted rCAR‐T therapy for antibody‐mediated autoimmune diseases.

**Methods:**

In the Phase 2a part of the study, Descartes‐08 was administered at 52.5 × 10^6^ CAR+ cells/kg per infusion with varying dosing frequencies as an outpatient treatment and without lymphodepletion chemotherapy. A subset of participants received Descartes‐08 as six weekly infusions and were followed long term with assessments conducted at 2, 3, 6, 9, and 12 months.

**Results:**

All seven participants who received six weekly infusions of Descartes‐08 exhibited clinically meaningful improvement in common MG severity scales (MG Composite, MG Activities of Daily Living, Quantitative MG scores, and Quality of Life 15‐revised) at Month 3 without significant toxicity. At Month 9 follow‐up, all participants continued to experience marked clinically meaningful improvements. Five out of seven participants maintained the response at Month 12. A third participant experienced a relapse approximately 6 months after completing on‐study follow‐up. All three participants who experienced loss of clinical effects were retreated. Two had rapid improvement in clinical scores with minimal symptom expression at Week 8, which was maintained through 12 months of retreatment follow‐up. The third participant experienced similar improvement in MG severity scores to their initial treatment.

**Interpretation:**

These data support continued development of Descartes‐08 in myasthenia gravis and other autoantibody‐associated autoimmune disorders.

## 1 Introduction

Generalized myasthenia gravis (MG) is an autoimmune disease in which antibodies against the neuromuscular junction—most commonly the nicotinic acetylcholine receptor (AChR)—trigger complement‐mediated damage and worsening muscle weakness and fatigue [1]. Up until 2017, mild forms of MG were treated with acetylcholinesterase inhibitors. Most patients with generalized MG require chronic treatment with glucocorticoids, nonsteroidal immunosuppressants, or thymectomy, and sustained disease control or remission is not consistently achieved [2]. More recently, biologics targeting complement protein C5 or the neonatal fragment crystallizable receptor (FcRn) have been used to prevent damage to the neuromuscular junction and to decrease circulating autoantibody levels, respectively [3]. However, both classes of drug require chronic administration and are associated with increased rates of infection, and some patients may have a suboptimal response, limiting their broad patient reach [4]. Targeting the immune system upstream of autoantibody production without resorting to broad immunosuppression has the potential to produce a more durable response after time‐limited treatment. Long‐lived plasma cells (LLPCs) residing in the bone marrow are the primary producers of the predominant anti‐AChR antibodies. In contrast, shorter‐lived plasmablasts have been associated with the production of antibodies directed against muscle‐specific kinase (MuSK), which account for approximately 10% of MG cases [5, 6]. Both types of cells express the B‐cell maturation antigen (BCMA), also known as tumor necrosis factor receptor superfamily member 17 (TNFRS17), making it a compelling target for the treatment of many antibody‐mediated diseases, including MG [7].

We recently reported on the safety and preliminary clinical activity of Descartes‐08 in patients with generalized MG (NCT04146051) [8]. Descartes‐08 is an autologous mRNA‐engineered chimeric antigen receptor (CAR) T‐cell therapy that targets BCMA on the surface of plasmablasts and mature plasma cells. Unlike conventional CAR‐T cell therapies that rely on in vivo T‐cell expansion to achieve therapeutic concentration, Descartes‐08 undergoes ex vivo T‐cell proliferation during manufacturing, enabling controlled dosing. This process allows Descartes‐08 to be administered in the outpatient setting and does not require lymphodepleting chemotherapy [9]. In the Phase 2a part of the study, all 7 participants (4 AChR Ab+, 2 MuSK Ab+, 1 triple [AChR, MuSK, low density lipoprotein receptor‐related protein 4] seronegative) who received six weekly infusions of Descartes‐08 demonstrated clinically meaningful improvements in common MG severity scales at Month 3, as defined by a reduction of at least 2 points on the MG Activities of Daily Living scale (MG‐ADL) and at least 3 points on both the MG Composite (MGC) and Quantitative MG (QMG) scales [10, 11, 12]. Three of the 7 participants achieved minimal symptom expression (MSE, defined as having MG‐ADL Score of 0 or 1) [13]. Two were intravenous immunoglobulin (IVIG) infusion‐dependent and one was plasmapheresis‐dependent for years prior to treatment, and all maintained responses without further IVIG or plasmapheresis. There were no instances of cytopenia, treatment‐associated infections, hypogammaglobulinemia, or depletion of protective vaccine titers such as the anti‐meningococcal and anti‐tetanus antibodies.

While these results demonstrated preliminary safety and clinical activity of Descartes‐08 in MG at 3 months, important remaining questions are the durability of response without ongoing treatment, the feasibility of retreatment for any subset of patients who experience worsening of disease, and the long‐term effect of BCMA‐directed mRNA CAR‐T therapy on immunoglobulin levels and vaccine titers. Here, we report the results of both the final, 12‐month follow‐up analyses and the optional retreatment of participants who met protocol‐specific retreatment criteria.

## 2 Methods

Clinical trial design, objectives, outcome measures, laboratory and data analysis methods, and demographic characteristics were previously described [8]. Briefly, this was a multicenter, open‐label, Phase 1/2a study conducted across eight academic centers and community neurology clinics in the United States. The study was conducted according to the International Conference on Harmonization of Good Clinical Practice, the principles of the Declaration of Helsinki, and applicable local ethical and legal requirements. Independent ethics committees and institutional review boards provided written approval for the study protocol and all amendments, and an independent study monitoring committee periodically reviewed and evaluated the accumulated study data for participant safety, study conduct, and study progress. All participants provided written informed consent.

**FIGURE 1:**
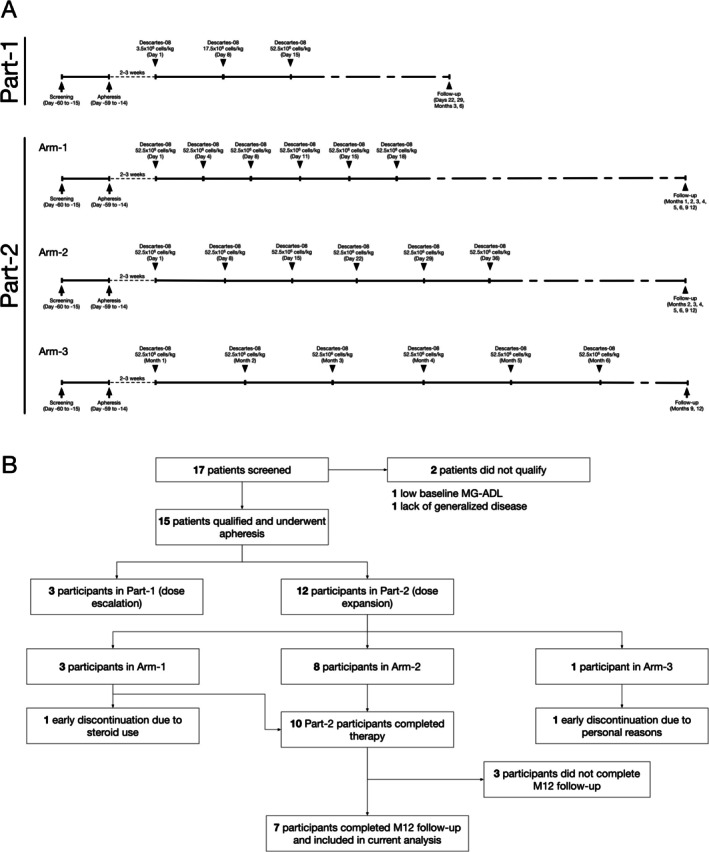
MG‐001 study schema (A) and CONSORT diagram (B).

Participants underwent leukapheresis to isolate peripheral blood mononuclear cells (PBMCs), which were subsequently processed and manufactured into Descartes‐08 under good manufacturing practice (GMP) guidelines. In Part‐1 of the study, three participants with Myasthenia Gravis Foundation of America (MGFA) class III–IV AChR‐antibody positive (AChR‐Ab+) generalized MG received escalating doses of Descartes‐08 intravenously over 30 min in an inpatient hospital setting. In Part‐2, up to 10 participants per arm with MGFA Class II–IV AChR‐Ab+, MuSK‐Ab+, or seronegative MG received the maximum administered dose of Descartes‐08 from Part‐1—which was 52.5 × 10^6^ viable CAR+ cells per kilogram—twice‐weekly for 3 weeks (Arm‐1), once‐weekly for 6 weeks (Arm‐2) or once‐monthly for 6 months (Arm‐3). Descartes‐08 was administered in an outpatient setting with 2 h of post‐infusion monitoring and without lymphodepletion chemotherapy (Figure 1A).

All participants had to be on stable doses of prednisone (up to 40 mg) or nonsteroidal immunosuppressants (methotrexate, azathioprine, or mycophenolate mofetil) for 8 weeks prior to enrollment. Tapering of the prednisone dose was allowed following completion of infusions; changes to the dose of nonsteroidal immunosuppressants and initiation of new MG‐specific medications were not allowed. In both parts, the participants received acetaminophen and diphenhydramine as premedication.

Participants were followed during infusions and at prespecified post‐infusion timepoints. The group that received Descartes‐08 as six weekly infusions (Arm‐2) had the longest follow‐up, with evaluations at Months 2, 3, 4, 5, 6, 9, and 12 counting from the first infusion, and was therefore included in this analysis. MG severity was assessed at each of those visits using the MG‐ADL [10], MGC [11], QMG score [12], and MG quality of life 15‐revised score [14] (QoL‐15r). MG‐ADL is a validated 8‐item, 24‐point, patient‐reported scale that assesses the impact of myasthenia symptoms on daily functioning [10]. QMG is a validated, standardized, quantitative, 39‐point strength scoring system consisting of 13 provider‐assessed items [12, 15]. MGC is a 10‐item, 60‐point weighted instrument composed of selected components of the MG‐ADL and QMG scores [11]. MG‐QoL‐15r is a 15‐item, 30‐point quality‐of‐life, patient‐reported instrument [14]. The raters were certified in administering these scales. Serum anti‐AChR antibody levels were measured by the central lab at follow‐up for each participant who had detectable levels at baseline. Serum anti‐MuSK antibody levels were measured by the local clinical site lab, where available.

A bead‐based multiplex immunoassay was used to measure the serum cytokines IL‐1β, IL‐2, IL‐4, IL‐6, IL‐10, IL‐12p70, IL‐17A, CCL2, CXCL8, CXCL10, TNF‐α, TGF‐β, and IFN‐γ (LEGENDplex™ human Essential Immune Response Panel [13‐plex] (BioLegend)). The assay was performed with serum diluted 1:2 according to the manufacturer's instructions. Data analysis was performed using the LEGENDplex™ Data Analysis Software (BioLegend), which calculates cytokine concentrations based on standard curves generated from serial dilutions of recombinant cytokines provided in the kit.

The levels of B‐cell Activating Factor (BAFF, TNFSF13B, or CD257) and A Proliferation‐Inducing Ligand (APRIL, TNFSF13, or CD256) in the serum samples were measured using enzyme‐linked immunosorbent assay (ELISA) kits. The BAFF ELISA is a validated Quantikine assay (R&D Systems, Minneapolis, MN, USA). The APRIL ELISA was from ThermoFisher (Waltham, MA, USA). Both assays were used according to the manufacturer's instructions. The optical density of each well was read at 450 nm using a microplate reader (BioTek Instruments, Winooski, VT, USA). The standard curves were generated by plotting the optical density values against the known concentrations of BAFF and APRIL. The concentrations of BAFF and APRIL in the serum samples were calculated by interpolating the optical density values from the standard curves. The results were expressed as pg/mL of BAFF and APRIL.

Numerical outcomes were expressed as mean change with a 95% confidence interval. We presented individual data points when n was 3 or fewer.

## 3 Results

As previously described, Arm‐1 and Arm‐3 participants discontinued study participation before the full protocol‐specified follow‐up period (Figure 1B) [8]. Seven patients were enrolled in Arm‐2, of whom 5 were females, 4 (57%) were AChR‐Ab+, 3 (43%) had previously undergone thymectomy, and all had received previous immunosuppressive treatments and IVIG. Most participants continued the use of pyridostigmine and corticosteroids throughout the study. All seven Arm‐2 participants completed 12 months of follow‐up, and the findings are reported here (Figure 1B). Descartes‐08 maintained a favorable safety profile, with no new product‐related adverse events reported during the 12‐month follow‐up period. Five of the 7 participants received prednisone at baseline, with a median daily dose of 17.5 mg. By Month 12, the median dose decreased to 5 mg daily. Neither of the two participants who were receiving IVIG once and twice monthly at baseline required IVIG during the 12 months of follow‐up.

**FIGURE 2:**
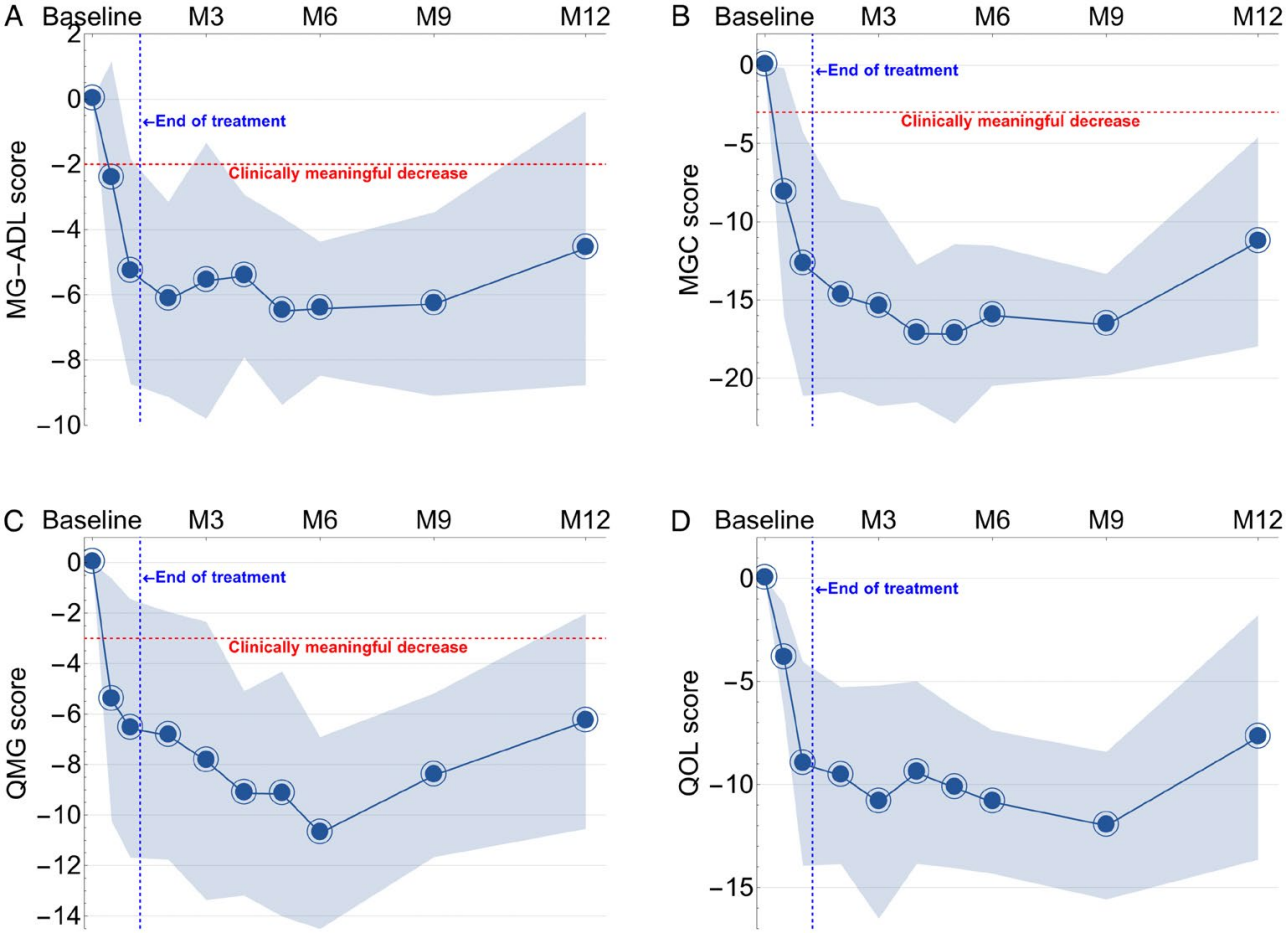
Changes in myasthenia gravis disease severity scores (blue line: mean, band: 95% confidence interval).

At Month 9, approximately 7 months after the last infusion and without new or increased MG‐directed drugs, all participants continued to experience substantial clinical responses as measured by MG‐ADL (mean change −6.3 [95% CI −3.5 to −9.1], Figure 2A), MGC (−16.6 [−13.4 to −19.5], Figure 2B), QMG (−8.4 [−5.2 to −11.6], Figure 2C), and QoL‐15r (−12 [−8 to −16], Figure 2D). At Month 12, 5 of the 7 participants maintained clinically meaningful improvement, including one with MSE, with mean change in MG‐ADL −4.6 [95% CI −8.8 to −0.4], MGC −11.3 [−18.0 to −4.6], QMG −6.3 [−10.5 to −2.0] and QoL‐15r −7.7 [−15.6 to −8.4]. Two participants with anti‐AChR antibody‐positive MG experienced worsening disease at Month 12 and became eligible for retreatment (Table S1); both opted for retreatment. At Month 18 after initial treatment, a third participant, with triple‐seronegative MG, experienced MG worsening and opted for retreatment.

**FIGURE 3:**
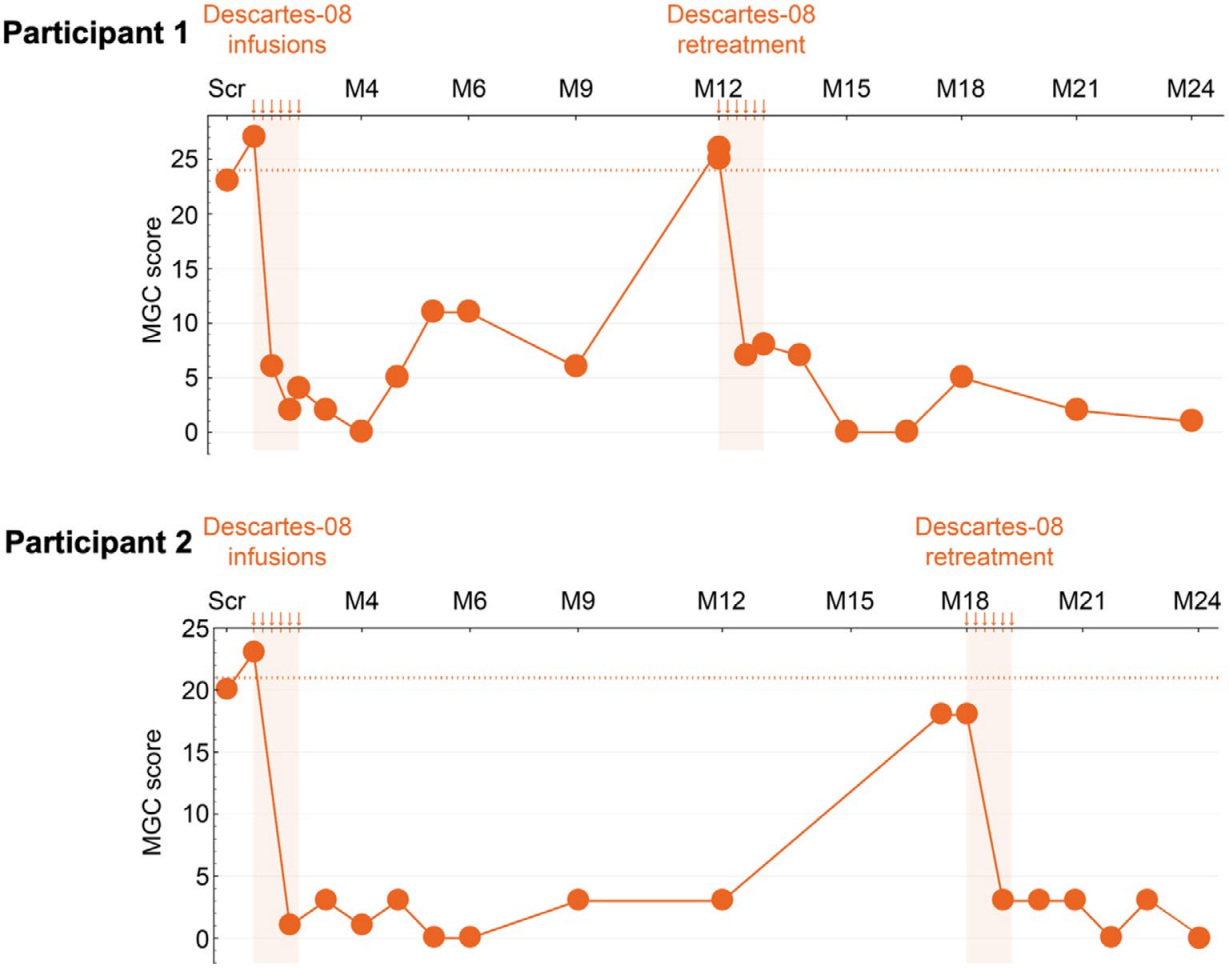
Change from baseline in MGC Score after initial dosing and retreatment in participants experiencing relapse at Month 12 and Month 18.

Retreatment consisted of repeat apheresis followed by six once‐weekly infusions of target 52.5 × 10^6^ viable CAR+ cells per kg in an outpatient setting and without lymphodepletion. The safety profile was unchanged compared to the initial treatment. All three participants experienced a rapid improvement in MG‐specific clinical scores; two achieved MSE by Week 8, which was maintained through 12 months of post retreatment follow‐up (Figure 3).

**FIGURE 4:**
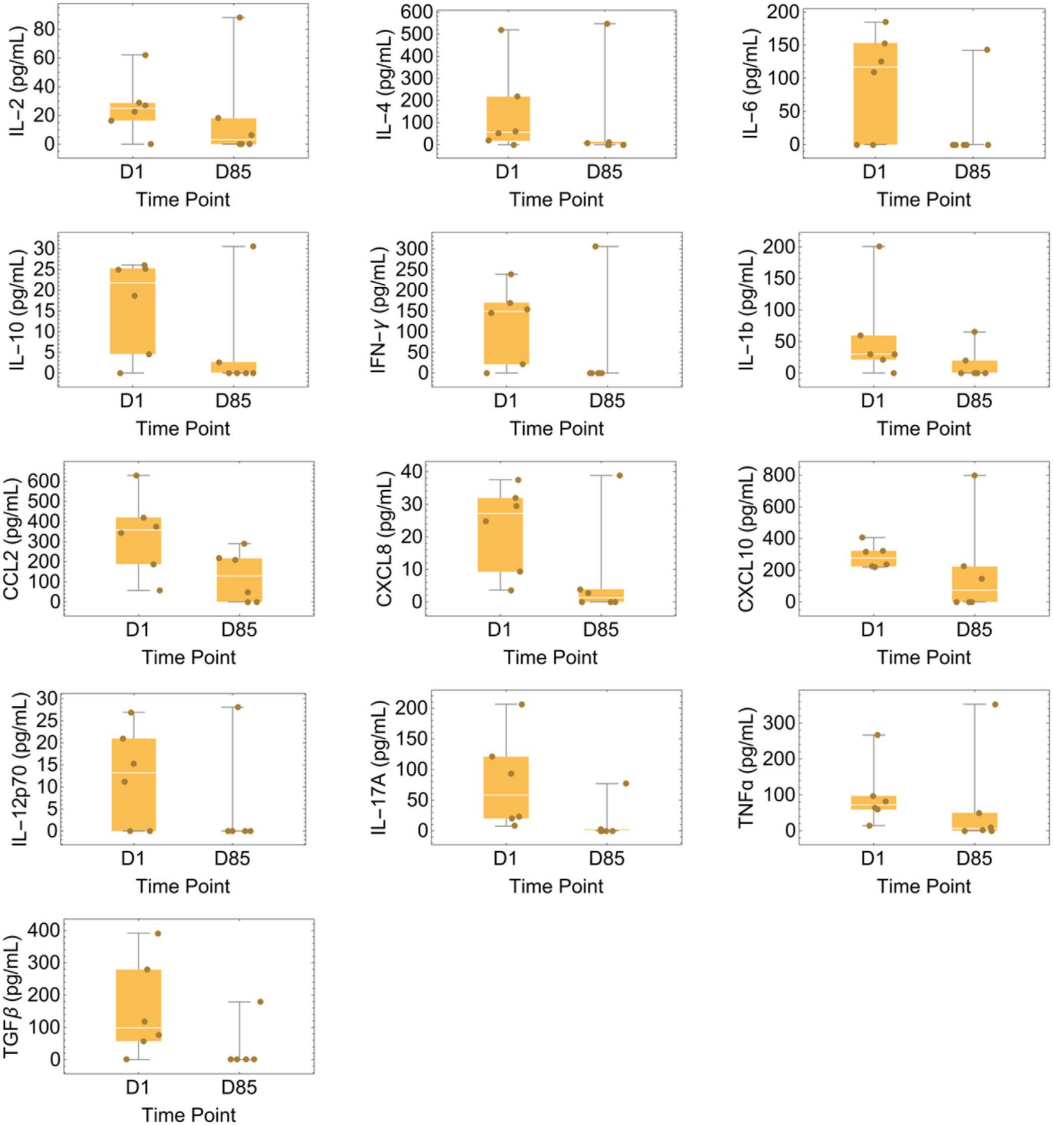
Serum cytokine levels pretreatment (“D1”) and at Month 3 after initiation of treatment (“D85”) (white line: median, orange box: interquartile range, bars: minimum and maximum values).

At baseline, all serum cytokines measured were below the level of quantification in one of the seven participants. In the other remaining six, median serum cytokine levels were decreased at Month 3 (Day 85) compared to pretreatment levels (Figure 4). Notably, the median level of interleukin‐17A decreased from 58.2 pg/mL pretreatment to 0 pg/mL at Month 3 (*p* = 0.018 by Mann–Whitney U‐test). Though values for most other measured cytokines also decreased, this was not statistically significant.

**FIGURE 5:**
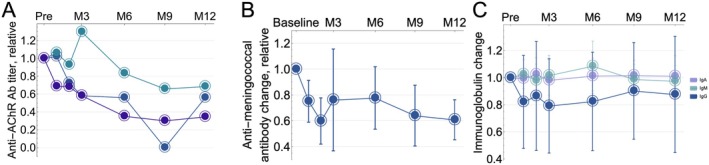
Changes in serum antibody levels over 12 months of follow‐up (A: Each color line: relative change from baseline for each participant with detectable antibodies at baseline. B: Blue line: mean, bars: 95% confidence interval. C: Each color line: mean [IgE was below the lower limit of quantification in all participants across all time points], bars: 95% confidence interval).

**FIGURE 6:**
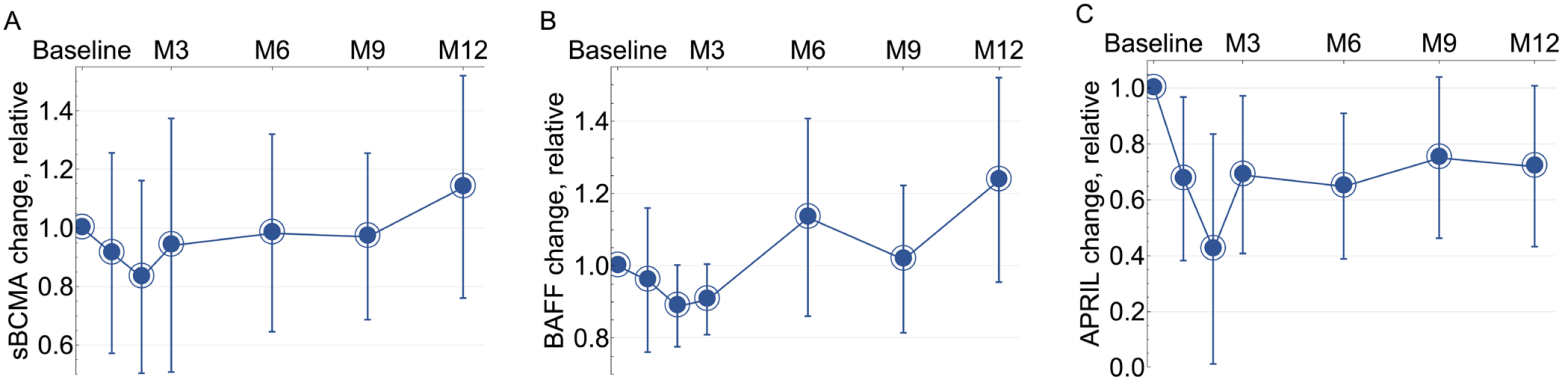
Changes in sBCMA, BAFF, and APRIL levels over 12 months of follow‐up (blue line: mean, bars: 95% confidence interval).

Three of the four participants with reported anti‐AChR antibody exhibited detectable levels at baseline, and all three showed reductions in antibody levels by Month 6 (−17%, −44%, and −65%). These reductions continued at Month 9 (−35%, −100% [undetectable], and −70%), and persisted at Month 12 (−33%, −44% and −65% reductions, Figure 5A). The participant whose anti‐AChR antibody was undetectable at Month 9 (< 0.3 nmol/L) and increased to 0.51 nmol/L at Month 12 also had worsening disease from Month 9 to Month 12 but did not opt for retreatment. A participant with anti‐MuSK antibody‐positive MG had a 25% reduction in serum antibody levels at Month 2 compared to pretreatment levels, as measured by the local lab. No other time points were available. Five of the seven participants had circulating anti‐meningococcal antibodies at baseline, all from prior vaccination. There was a small but detectable decrease in these antibodies at Month 3, which deepened by Month 9 (−48% [−25% to −75%]) and stabilized by Month 12 to lower but still protective levels (−48% [−35% to −69%], Figure 5B), following a similar pattern to the anti‐AChR antibody reduction. In contrast, there was no appreciable decrease in total immunoglobulin (Figure 5C), other vaccine‐associated antibodies (Figure S1), or soluble BCMA (sBCMA, Figure 6A), indicating that the targeting of BCMA‐positive plasma cells was not indiscriminate. Interestingly, both B‐cell growth factors APRIL and BAFF had a small but detectable decrease at Month 3 (APRIL −31% [−3% to −59%], BAFF −9% [−19% to 1%], Figure 6B,C). The decrease in APRIL seemed to have persisted at Month 12 (−28% [−57% to 1%], Figure 6C).

## 4 Discussion

The initial report of Descartes‐08 noted deep responses across MG types and degrees of severity [8]. In this 12‐month follow‐up, we observed durable responses sustained up to 12 months in 5 of the 7 (71%) participants who received six once‐weekly doses. The responses continued beyond 12 months; retreatment demonstrated clinical activity similar to or better than the initial treatment course. The durability of response parallels the reduction of anti‐AChR antibody titers in participants with detectable levels at baseline; response may be extended further with retreatment for patients who experience worsening of symptoms without affecting the overall favorable safety profile. Notably, cytokine profiling trended toward reductions in several pro‐inflammatory cytokines with no evidence of cytokine release syndrome (CRS). The clinical effect appeared sustained for up to a year without any ongoing treatment beyond the initial 6‐week course of outpatient infusions. Thus, our study highlights the potential of mRNA cell therapy to transform the treatment paradigm for MG and other autoimmune diseases, shifting from a chronic immunosuppressive therapy to potentially one‐time or as‐needed treatments. Such advances would bring durable responses without increasing the risk of opportunistic infection, as well as more consistent disease control that mitigates the periodic waves of disease flair and control associated with existing therapies relying on ongoing treatment.

One open question for mRNA‐based therapies is the feasibility of repeat infusions with these technologies. Initial studies of a mesothelin‐directed mRNA CAR‐T for treatment of cancer reported anaphylactic reactions tied to the use of a murine CAR construct [16]. In contrast, there were no cases of anaphylaxis reported across 14 patients treated and 88 administered infusions with Descartes‐08 in MG, including in 3 participants who received a total of 12 infusions each. Notably, the three participants reported fewer adverse events after retreatment while experiencing the same or better numeric improvement across MG severity scales all the way to MSE. As with any form of biologic therapy, allergic reactions, including anaphylaxis, cannot be excluded. Indeed, a participant with a history of drug‐induced urticaria was enrolled to Arm‐1 (twice‐weekly dosing) and experienced grade 3 urticaria after the third infusion of Descartes‐08. However, the cumulative safety data indicate a low rate of allergic reactions, with no anaphylaxis to date.

Additionally, the use of mRNA for autologous CAR T‐cell engineering may decrease the risk of several toxicities commonly associated with CAR‐Ts engineered with integrating vectors. As there is no lymphodepletion chemotherapy required, there were no associated hematologic toxicities in any of the 14 patients with MG who received Descartes‐08. In contrast, all 16 patients with various autoimmune disorders (8 with systemic lupus erythematosus, 4 with systemic sclerosis, 3 with idiopathic inflammatory myositis, and 1 with MG) who received conventional CAR‐Ts directed toward CD19 under an expanded access (“compassionate use”) protocol had severe (grade ≥ 3) neutropenia lasting median 14–16 days [17, 18]. Five participants had infections within 3 months of treatment, and all but 3 had at least one infection through 12 months of follow‐up.

The second common group of toxicities for conventional CAR‐T therapies is CRS and immune effector cell‐associated neurotoxicity syndrome (ICANS), which are thought to be the result of hyper‐proliferation of CAR‐Ts and an excess of cytokine secretion [19]. In the same compassionate use case series, 12 patients (75%) developed CRS, 1 (6%) developed ICANS, and 6 (37.5%) received tocilizumab, the anti‐IL6 monoclonal antibody commonly used to treat CRS. No cases of CRS or ICANS were observed among the 14 open‐label MG‐001 participants dosed with Descartes‐08, and no reported use of tocilizumab or corticosteroid. These findings align with our initial hypothesis that mRNA CAR‐T therapy presents potentially a lower risk for CRS or ICANS due to transient expression of the CARs, resulting in a progressive decline in CAR concentration as T‐cell proliferation subsides.

The third concern is genomic integration inherent to the use of lentiviral vectors in the production of conventional CAR‐Ts, which in some cases may have led to the development of incurable T‐cell lymphoma and death of patients receiving CAR‐Ts for treatment of other hematologic malignancies [20]. This complication, paired with the already known risk of secondary malignancies associated with alkylating agents such as cyclophosphamide used for lymphodepletion, has led to requirements for prolonged, 15‐year monitoring of patients receiving conventional CAR‐Ts. mRNA is not associated with genomic integration, and no cases of malignancies due to other mRNA‐based interventions such as vaccines have been reported. There is therefore no requirement for long‐term monitoring after treatment with mRNA CAR‐Ts.

Descartes‐08 targets BCMA, which is uniquely expressed in two groups of cells: (1) B cells differentiated toward antibody secretion such as plasmablasts and long‐lived plasma cells, and (2) plasmacytoid dendritic cells (pDCs) of the myeloid lineage. Plasma cells are the source cells that secrete pathogenic antibodies during MG and many other autoimmune diseases, while pDCs play a key role in type 1 interferons (e.g., interferon‐α) involved in immune (and autoimmune) responses. The pattern of anti‐AChR antibody reduction observed in patients with detectable levels at baseline, with a slow tapering over several months after the last Descartes‐08 infusion and peak reduction after Month 6, approximately 4.5 months after the last dose, is consistent with the mechanism of action removing the autoantibody source—the plasma cell—without a direct effect on the pool of circulating antibodies. The sustained numerical improvement in MG severity scores may therefore be explained by this durable decrease in autoantibody levels. However, the onset of clinical improvement was more rapid than the autoantibody reduction, and circulating antibody titers may not accurately reflect their concentration at the tissue level. Previous studies in MG have yielded inconsistent findings regarding the utility of anti‐AChR antibody levels as biomarkers in MG, with limited reliability in monitoring disease severity or predicting treatment response [21, 22]. Another possible reason is the effect of Descartes‐08 targeting BCMA‐positive pDCs, which may lead to a reduction of the pro‐inflammatory cytokines necessary for the full manifestation of the pathogenic effect of antibodies.

Notably, total immunoglobulin levels did not significantly decrease, and of all vaccines tested, only the meningococcal titer decreased while maintaining protective levels. In line with this finding and as previously reported, there was minimal change in the serum levels of soluble BCMA. An analysis of body‐wide expression of BCMA showed that mucosal and connective tissue‐associated plasma cells account for the bulk of BCMA‐expressing cells. Unless given a specific homing signal, T cells, including CAR‐T cells, are not expected to traffic in these tissues to the extent that they do in circulation, lymphoid organs, and the bone marrow. A preference toward BCMA‐directed CAR‐Ts eliminating BCMA‐expressing cells in circulation (short‐lived plasmablasts) and the bone marrow (long‐lived plasma cells) would therefore not be unusual. Incidentally, these two populations are known to secrete pathogenic MuSK and AChR antibody.

Several lines of evidence indicate that Descartes‐08 may be a disease‐modifying treatment rather than symptomatic therapy. The first is the durability of the apparent treatment effect well beyond the last dose of administered cells, with participants showing clinically meaningful improvement in disease severity scores—including MSE—months after treatment completion. The second is the decrease in anti‐AChR antibody levels associated with treatment, despite Descartes‐08 not acting at the level of immunoglobulins directly. Lastly, retreatment with Descartes‐08 in participants who had initial improvement followed by worsening at 12–18 months resulted in a greater and more durable response in at least one participant. This observation suggests the existence of residual pathogenic BCMA‐positive cells that could have contributed to disease relapse, but they were easier to eradicate with retreatment compared to the larger pool of cells targeted with the initial treatment. More experience with initial treatment and retreatment would be needed to confirm this hypothesis.

In summary, we observed continued clinical improvement and autoantibody reductions after BCMA‐directed mRNA CAR‐T treatment that persisted through the 1‐year follow‐up period. Patients with clinical relapses at Month 12 and Month 18 again achieved MSE after retreatment with six additional once‐weekly infusions. The favorable safety profile of Descartes‐08 contrasts with DNA‐based CAR‐Ts, which carry oncogenic risk from genomic integration of CAR DNA and require lymphodepletion chemotherapy with potential hematologic toxicities. Our observations suggest that using mRNA CAR‐Ts to target BCMA can result in durable depletion of autoantibodies and clinically meaningful improvement in MG severity scores without severe toxicity, for example, agammaglobulinemia, or increased risk of infection. These data support continued development of Descartes‐08 in MG and other autoimmune disorders.

## Author Contributions

M.K., M.D.M., and T.M. contributed to the conception and design of the study; N.C., G.S., M.H.F., T.V., J.F.H., T.M., M.D.M., C.A.S., and C.M.J. contributed to the acquisition and analysis of the data; M.D.M., J.F.H., T.V., and C.A.S. contributed to drafting the text or preparing the figures.

## Conflicts of Interest

Nizar Chahin has received honoraria as a consultant or advisory board member from argenx, Sanofi, Amicus, and Sarepta. Gregory Sahagian: consulting fees from UCB Biosciences, Immunovant; honoraria from argenx, Alexion; travel support from argenx, Immunovant. Marc H. Feinberg: honoraria from argenx. Christopher M. Jewell: equity in Barinthus Biotherapeutics and Nodal Therapeutics; employee: the University of Maryland and VA Maryland Health Care System. The views in this paper do not reflect the views of the state of Maryland or the US Government. C. Andrew Stewart, Christopher M. Jewell, Metin Kurtoglu, and Miloš D. Miljković: employees, ownership interest in Cartesian Therapeutics. Tuan Vu: honoraria from Alexion, argenx, Dianthus, ImmunAbs, Johnson & Johnson, NMD Pharma, and UCB Biosciences. Tahseen Mozaffar: consulting fees from Alexion Pharmaceuticals Inc., Amicus, Annji, argenx, Audentes/Astellas Gene Therapy, Horizon Therapeutics, Maze Therapeutics, Momenta, Sanofi, UCB; support for attending meetings and/or travel from Sanofi; participation on a DSMB or an advisory board from Sarepta, Applied Therapeutics, and the National Institutes of Health. James F. Howard Jr.: consulting fees from Alexion AstraZeneca Rare Disease, Amgen, argenx, Biohaven Ltd., F. Hoffmann‐LaRoche Ltd., Hansa Biopharma, Merck EMB Serono, NMD Pharma, Novartis Pharma, Regeneron Pharmaceuticals Inc., Sanofi US, Seismic Therapeutics, UCB Biosciences. All other authors declare no competing interests.

## Supporting information


**Figure S1.** Vaccine‐associated antibody levels for individual participants who received six once‐weekly infusions of Descartes‐08.
**Table S1.** Baseline characteristics of participants who received retreatment with Descartes‐08.

## Data Availability

Access to anonymized, individual, and trial‐level data (analysis datasets) will be provided by request from qualified researchers performing independent, rigorous research, after review and approval of a research proposal and statistical analysis plan and execution of a data sharing agreement. Data requests can be submitted at any time; the data will be accessible for 12 months. Requests can be submitted to trials@cartesiantx.com.
