# Supplementary material for: Durability of Response to B‐Cell Maturation Antigen‐Directed mRNA Cell Therapy in Myasthenia Gravis
Source: Ann Clin Transl Neurol. 2025 Aug 26;12(11):2358–66. doi: 10.1002/acn3.70167 (PMC12623823; doi:10.1002/acn3.70167)
Supplement: Supplementary file 1 — Figure S1. Vaccine‐associated antibody levels for individual participants who received six once‐weekly infusions of Descartes‐08. Table S1. Baseline characteristics of participants who received retreatment with Descartes‐08. [file ACN3-12-2358-s001.docx]

Safety and Efficacy of Autologous RNA Chimeric Antigen Receptor T-cell (rCAR-T) Therapy in Myasthenia Gravis

Supplementary Appendix

# Table of Contents

MG-001 Study Team members 2

Supplementary Figures

Figure s1: Vaccine-associated antibody levels for individual participants who
received six once-weekly infusions of Descartes-08. 3

Supplementary Tables

Table s1: Baseline characteristics of participants who received retreatment
with Descartes-08. 4

# MG-001 Study Team members

Volkan Granit MD^1^, Michael Benatar MD PhD, ^1^ Tahseen Mozaffar MBBS^2^, Nizar Chahin MD^3^, James F. Howard Jr. MD^4^, Adam D. Slanksy MD^5^, Marc H. Feinberg MD^6^, Gregory Sahagian MD^7^, Tuan Vu MD^8^, Denise Pereira MD^1^, Julie Steele RN^1^, Maria Elena Paredes MD^1^, Cara Benjamin PhD^1^, Ali Aamer Habib MD^2^, Julia Kimberly Fong CRC^2^, Luis De La Cruz CRC, ^2^ Diana Dimitrova PhD^3^, Manisha Chopra MBBS CCRP^4^, Kelly Holley CRC^5^, Gabrielle DeMaria MS^6^, April Tenorio CCRC^7^, Naraly Requena^8^, Beverly “Mackenzie” Brooks^8^, Niraja Suresh MD^8^, Jerrica Farias ARNP^8^, Miloš D. Miljković MD MSc^9^, Metin Kurtoglu MD PhD^9^, Minhtran Ngo Casi MD^9^, Adam Chowdhury MD^9^, C. Andrew Stewart PhD^9^, Mehmet Tosun MD^9^, Yufei Shang PhD^9^, Matthew T. Duverney PhD^9^, Maria Kireeva PhD^9^, Emily English PhD^9^, Christopher Jewell PhD^9^, Michael Singer MD PhD^9^, Murat Kalayoglu MD PhD^9^.

^1^ University of Miami, Florida
^2^ University of California Irvine
^3^ Oregon Health and Sciences University, Portland
^4^ University of North Carolina at Chapel Hill
^5^ Neurology Associates, Orlando, Florida
^6^ SFM Research, Boca Raton, Florida
^7^ Neurology Center of Southern California, San Diego, California
^8^ University of South Florida, Tampa
^9^ Cartesian Therapeutics, Fredrick, Maryland

**F****igure s1.** **Vaccine-associated antibody levels for individual participants who received six once-weekly infusions of Descartes-08.** Early: Day 1, 8 or 15; Mid: Month 2, 3 or 4; Late: Month 12. Participant 2 was retreated and designated as “2R” after retreatment. Red X denotes time points for which a sample was not available.


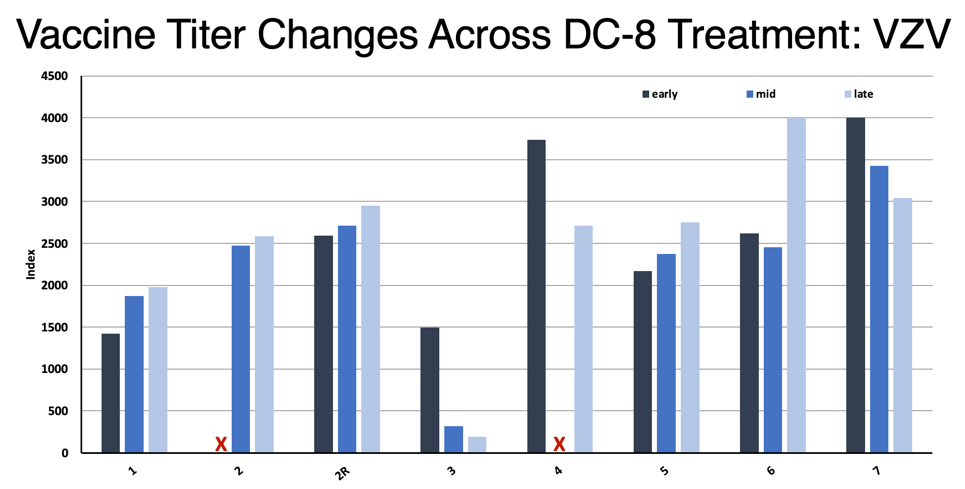

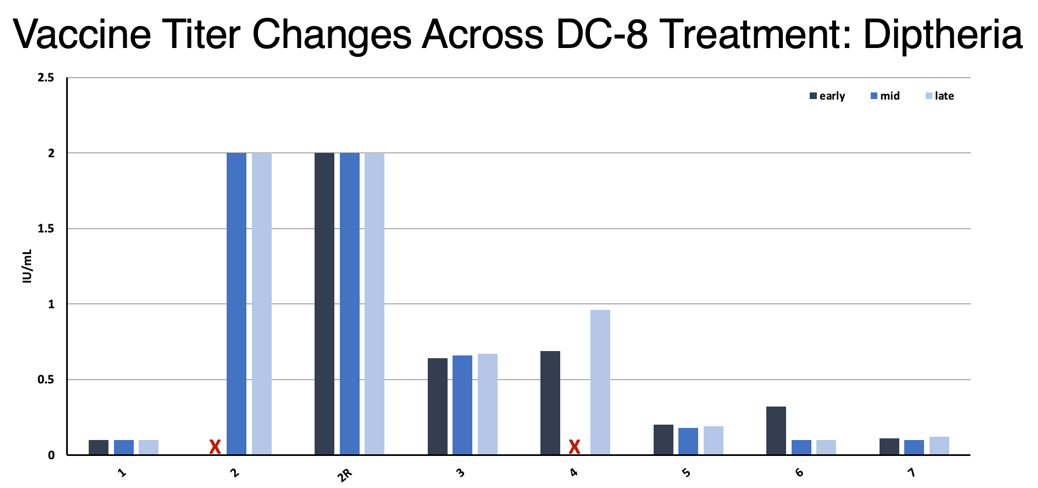


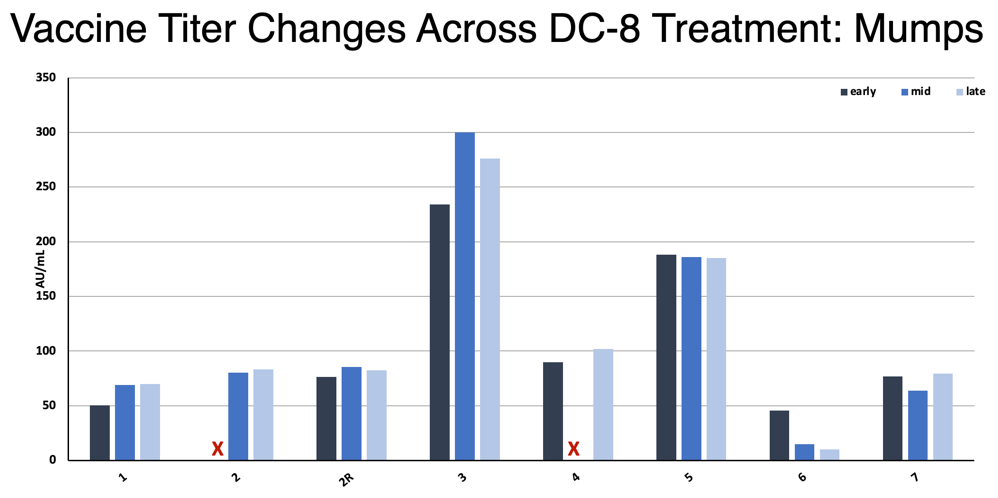

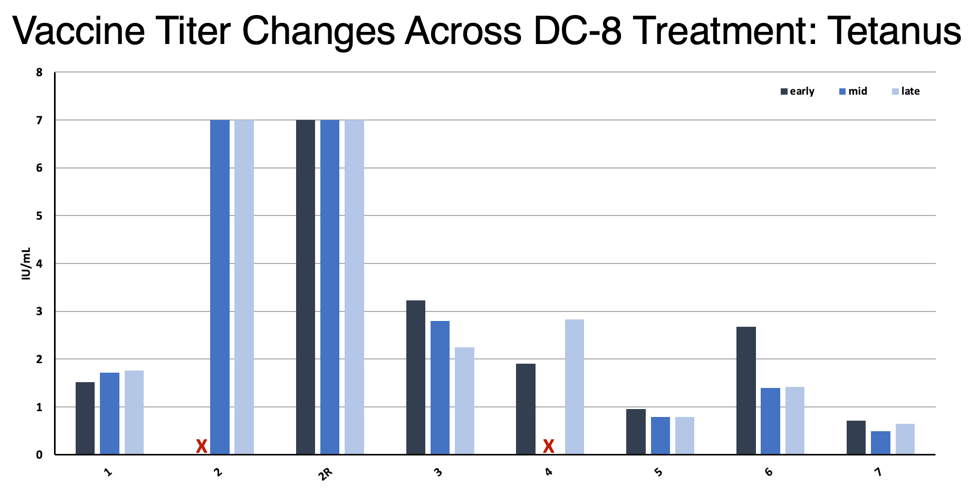


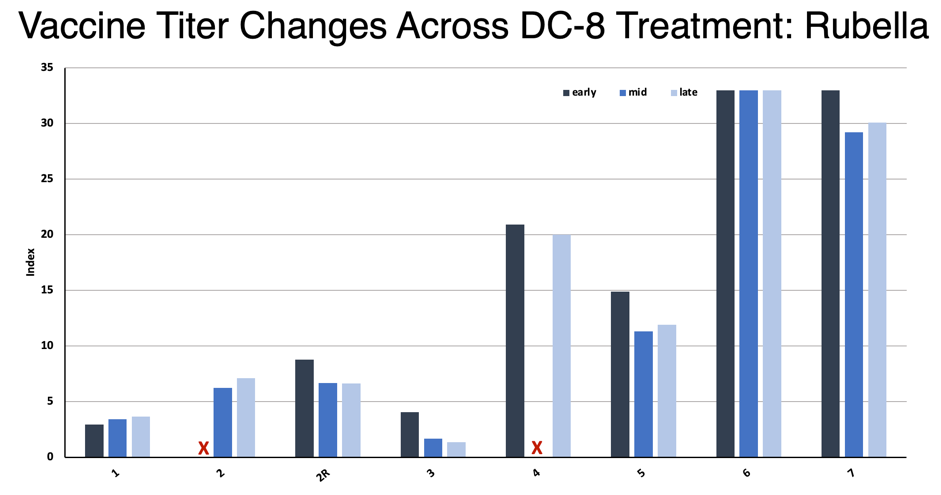

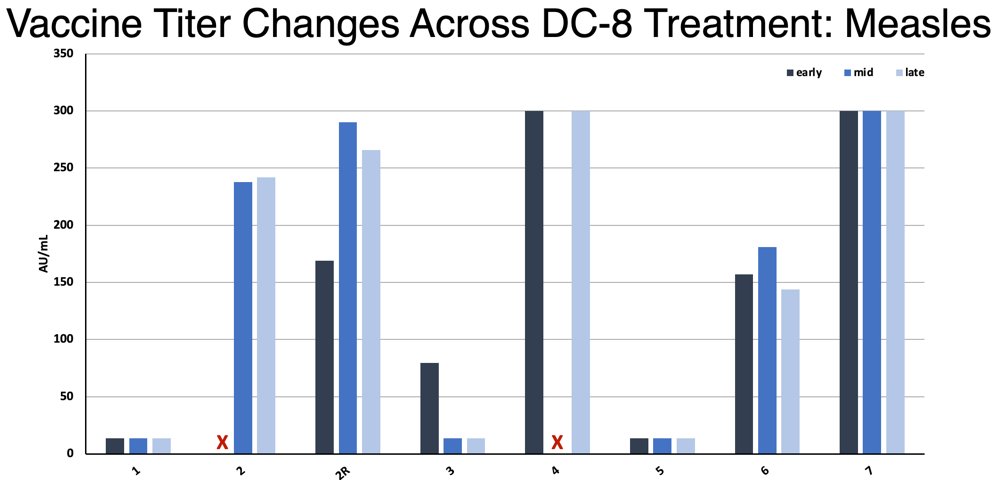


**Table s1.** **Baseline characteristics of participants who received retreatment with Descartes-08.**

| No. | Age | Sex | Weight (kg) | Ab | MGFA Class | Age at onset | MG duration (years) | Baseline MG severity scores | | | | Ongoing/ most recent treatment | Actual dose per infusion (CAR+ cells/kg, ⨉ 10^6^) | Number of infusions | Total infused CAR+ cells  (⨉ 10^9^) |
| --- | --- | --- | --- | --- | --- | --- | --- | --- | --- | --- | --- | --- | --- | --- | --- |
|  |  |  |  |  |  |  |  | QMG | MGC | MG ADL | QoL 15r |  |  |  |  |
| 1 | 52 | F | 110.0 | AChR | IIa | 36 | 16 | 16 | 27 | 13 | 26 | Pyr, Aza | 22.7* | 6 | 11.34 |
| 2 | 50 | F | 81.5 | AChR | IIIa | 26 | 23 | 23 | 26 | 12 | 22 | IVIg, MMF, Pyr, Aza | 63.1 | 6 | 30.85 |
| 3 | 66 | M | 88.0 | Other | IIa | 57 | 10 | 19 | 23 | 8 | 14 | Pyr, Pred | 30.0 | 6 | 15.62 |

*Actual dose was lower than the 52.5x10^6^±45% target
